# Sex-Specific Causes and Consequences of White Matter Damage in a Middle-Aged Cohort

**DOI:** 10.3389/fnagi.2022.810296

**Published:** 2022-05-11

**Authors:** Nadine Bonberg, Niklas Wulms, Mahboobeh Dehghan-Nayyeri, Klaus Berger, Heike Minnerup

**Affiliations:** ^1^Institute of Epidemiology and Social Medicine, University of Münster, Münster, Germany; ^2^Clinic of Radiology, Medical Faculty, University Hospital Münster, University of Münster, Münster, Germany; ^3^Department of Psychosomatic Medicine and Psychotherapy, LVR Clinic, Medical Faculty of the Heinrich-Heine-University Düsseldorf, Düsseldorf, Germany

**Keywords:** sex, white matter damage, white matter hyperintensities (WMH), fractional anisotropy, vascular risk factors, cognition, cognitive reserve

## Abstract

**Objective:**

To evaluate potential sex-specific effects of multiple cardiovascular risk factors on white matter pathology in normal aging men and women, as well as potential sex-differences in the association of white matter pathology and cognitive functions.

**Methods:**

We analyzed cross-sectional data of 581 participants (median age: 53 years, 54% women) of the population-based cohort of the BiDirect Study who completed clinical examinations, five neuropsychological tests, and an 3T MRI examination. White matter pathology was determined by the extent of white matter hyperintensities (WMH) on FLAIR images as well as the magnitude of global fractional anisotropy (FA) based on diffusion tensor imaging. Main effects, interaction as well as sex-stratified generalized linear regression models were used to evaluate the moderating effect of sex on the association of hypertension, diabetes mellitus, smoking, and obesity with WMH and FA, respectively. Associations of imaging markers with cognitive test results were determined with linear regression models.

**Results:**

Hypertension showed stronger associations with more extensive WMH and less FA in women compared to men. Current smoking was associated with more severe WMH in women only. Adjusted for age and education, WMH were not significantly associated with cognitive tests, but higher FA was associated with better performance in motor function in both sexes and with executive functions in men, even after adjustment for cardiovascular risk factors.

**Conclusion:**

We observed a stronger association of hypertension and smoking with white matter damage in women, suggesting a higher susceptibility for vascular pathology in women. However, there was no association of WMH with cognition, and FA was associated with executive function tests only in men, suggesting a higher cognitive reserve in women.

## Introduction

White matter hyperintensities (WMH) on T2-weighted magnetic resonance images (MRI) are common in elderly people (Wen and Sachdev, 2004). Being strongly associated with vascular risk factors they are recognized as a marker of cerebral small vessel disease and as such with an increased risk of cognitive decline and dementia (Debette and Markus, 2010; Wardlaw et al., 2013; Kloppenborg et al., 2014).

Nevertheless, the etiology and pathogenesis of WMH is not well understood and most probably encompasses genetic as well as environmental factors (Gouw et al., 2011). Of those, sex seems one obvious factor as sex differences are known in the etiology of vascular disease (Förster et al., 2009; Fatemi et al., 2018) as well as in normal cognitive aging (McCarrey et al., 2016) and the development of pathological cognitive decline (Li et al., 2014). Men usually show a higher prevalence of cardiovascular risk factors (Fatemi et al., 2018), whereas there is increasing evidence that women have higher WMH volumes (Leeuw et al., 2002; van den Heuvel et al., 2004; Wen and Sachdev, 2004; Sachdev et al., 2009; Fatemi et al., 2018), and sex differences in cognition generally tend toward a better cognitive performance in women (McCarrey et al., 2016; Reas et al., 2017; Levine et al., 2021; Nichols et al., 2021). Given that WMH are presumed to mediate part of the association between cardiovascular risk factors and cognitive performance (Chen et al., 2021), a lower prevalence of vascular risk factors in women contrasting a higher burden of WMH suggests a higher susceptibility to white matter damage in women. Moreover, better cognitive performance despite a higher lesion load calls for investigations into sex-differences in cognitive and brain reserve (O’Dwyer et al., 2012; Stern et al., 2020; Levine et al., 2021; Subramaniapillai et al., 2021). Another approach to solving this apparent paradox might be the evaluation of alternative or more subtle markers of white matter damage. Diffusion tensor based measures, such as fractional anisotropy (FA) have been established as sensitive markers of brain and cognitive aging (Salat et al., 2005; Zavaliangos-Petropulu et al., 2019; Veldsman et al., 2020). Whereas WMH are defined by their macroscopic appearance on FLAIR-images and are highly heterogeneous regarding their underlying pathophysiology, FA reflects the microstructural integrity of the white matter (Le Bihan and Johansen-Berg, 2012) and might therefore be a more sensitive surrogate of risk-factor-associated damage (Wassenaar et al., 2019; Williams et al., 2019; Veldsman et al., 2020). Studies regarding sex-differences in diffusion tensor imaging (DTI) measures are inconsistent, mainly reporting higher FA in men (Inano et al., 2011; Kanaan et al., 2014; Takao et al., 2014).

Taken together, the moderating effect of sex in the association between modifiable risk factors and cognition is increasingly investigated. However, to our knowledge, there is only one study, that investigated sex-differences in cardiovascular risk factors, WMH and cognition simultaneously in a rather small cross-sectional study (Sachdev et al., 2009). The authors found different causes and consequences of white matter hyperintensities across sexes (Sachdev et al., 2009). We expand this multidimensional evidence by a larger cohort with additional evaluation of fractional anisotropy, and a broad battery of neuropsychological tests. The present study thus offers a broad perspective on sex-differences in white matter pathology based on a large community-dwelling cohort. First, the influence of multiple vascular risk factors on WMH and global FA is evaluated for middle-aged men and women. Second, the sex-specific impact of white matter pathology on several cognitive functions is analyzed, while considering potential mediating effects of vascular risk factors.

## Materials and Methods

### Subjects

We analyzed baseline data from 581 community-dwelling participants of the longitudinal BiDirect Study in Münster, Germany. All participants underwent a computer-guided interview, self-administered questionnaires, sensory and neuropsychological assessments, clinical examinations (e.g., anthropometry, vascular status, and blood sampling), as well as magnetic resonance imaging (MRI) of the brain (Teismann et al., 2014; Teuber et al., 2017). The data acquisition was conducted by a trained study team. For the present analysis, we applied several exclusion criteria. We excluded participants with clinical or imaging evidence of severe neurological disorders (stroke, Parkinson’s Disease, epilepsy, and multiple sclerosis), missing or invalid neuropsychological data including reduced German language skills, and missing or invalid MRI data, respectively. The BiDirect study was approved by the ethics committee of the University of Münster and the Westphalian Chamber of Physicians in Münster, North Rhine-Westphalia, Germany. All participants gave their written informed consent for study participation.

### Assessment of Education, Cardiovascular Risk Factors, and Depression

In personal interviews we assessed socio-demographic characteristics, participants’ smoking status, and participants’ health status and history. Education was documented in the four categories (1) primary or general secondary school, (2) intermediate secondary school, (3) high school and (4) university graduates. Current medications (e.g., hypertensive treatments) were recorded and body size and height as well as blood pressure were measured in a standardized way (Teismann et al., 2014). For analysis, uncontrolled blood pressure was defined as a systolic blood pressure ≥140 mmHg and/or a diastolic blood pressure ≥90 mmHg. A combination of current hypertensive treatment (yes/no) and measured blood pressure (controlled/uncontrolled) was used to define the categorical variable “arterial hypertension.” A history of diabetes was classified into “no physician diagnosis,” “diagnosed ≤7 years,” and “diagnosed >7 years”. Body mass index (BMI) was calculated from measured weight and height (kg/m^2^) and categorized into obesity (BMI ≥ 30 kg/m^2^) and no obesity (BMI < 30 kg/m^2^) according to the definition from the World Health Organization. The self-reported presence of depressive symptoms was measured by the Center for Epidemiological Studies Depression-Scale (CES-D) (Teismann et al., 2014) and categorized into CES-D score <16 (no clinically relevant depressive symptoms) and ≥16 (clinically relevant depressive symptoms) (Radloff, 1977). Smoking status was defined categorically as never vs. former vs. current smoking.

### Neuropsychological Assessment

The following five validated neuropsychological tests were administered to all participants:

(1) *Color-Word-Interference-Test (CWIT)*: A paper-pencil version with three task sets (words, color, and color-word) consisting of 36 items each, was administered to the participants and the reaction time was measured for each task set (Stroop, 1935). To measure interference control, a measure of working memory capacity, we calculated the interference time as the time difference of the second (color) and third (color-word) task set of the CWIT test.
(2) *Trail Making Test (TMT) A and B*: In TMT A, the time that was needed to connect consecutive numbers from 1 to 25 was recorded to measure attention and psychomotor speed. In TMT B, the time that was needed to connect consecutive numbers and letters in an alternating sequence (1-A-2-B-3-C, etc.) was recorded to measure working memory and mental set shifting (Reitan, 1992).
(3) *Regensburg Word Fluency Test (“animal naming test”)*: The number of animals that were named by the participants in 60 s were denoted to measure categorical association (semantic) fluency as a measure of executive function (Morris et al., 1989; Tombaugh et al., 1999).
(4) *Word List*: A recorded 12-item emotional word list was presented via loudspeaker two times to the participants. After each of the two presentations the participants were asked to reproduce as many words as possible. A third free recall followed after an interval of 15 min. This test was used to measure verbal retentiveness and memory (Kissler et al., 2006).
(5) *Purdue Pegboard Test*: The number of pegs that were placed by the participants in a wooden board within 30 s, first with the right hand, followed by the left hand, were used to measure fine motor skills (Tiffin and Asher, 1948).

A *Z*-score was calculated for each test or subtest using the respective test mean and standard deviation of the female subgroup for standardization. Test results from TMT A and B and the interference time from CWIT were log-transformed before standardization. All *Z*-scores were scaled, so that higher values represent better test results. Afterward, the scores for the three runs of the word list and the scores for the right and left hand from the Purdue Pegboard Test were averaged for analysis.

### Magnetic Resonance Imaging Acquisition

Magnetic resonance imaging was performed with the following protocol (Teuber et al., 2017) on the same 3.0 T MRI scanner (Intera with Achieva update; Philips Medical Systems, Best, Netherlands). The structural MRI protocol included a T1-weighted 3D TFE sequence with preceding inversion pulse, a T2 weighted FFE sequence, a TSE-FLAIR sequence, and a diffusion weighted sequence. The T1-weighted images were acquired in sagittal orientation with the following parameters: repetition time (TR)/echo time (TE) of 7.26/3.56 ms; flip angle of 9°; matrix size of 256 × 256; field of view (FOV) of 256 mm × 256 mm; reconstructed to pixel size of 1.00 mm × 1.00 mm; 160 slices with slice thickness of 2.0 mm with no gap between slices. The FLAIR images were acquired in axial orientation with TR/TE/TI of 11,000/120/2,600 ms; flip angle of 90°; matrix size of 352 × 206; FOV of 230 mm × 186 mm; reconstructed to pixel size of 0.45 mm × 0.45 mm; 27 slices with slice thickness of 4.0 mm with 1.0 mm gap. The diffusion weighted images were acquired with echo planar imaging (Single Shot SE-EPI, TR/TE = 5,900/95 ms); 36 slices with slice thickness of 3.6 mm with no gap, FOV of 240 mm × 240 mm, reconstructed to pixel size of 0.94 mm × 0.94 mm. Diffusion gradients were applied along 20 non-collinear directions at a *b* value of 1,000 s/mm^2^ and an additional undirected b0 image with 0 s/mm^2^.

### Image Analysis

Diffusion tensor imaging data were processed using the FSL v6.0.3 (Jenkinson et al., 2012) software library developed at the Oxford Centre for Functional MRI of the Brain (FMRIB). The pipeline of the PSMD marker tool provided at http://www.psmd-marker.com was used to process all images (Baykara et al., 2016). First, eddy current-induced distortion and motion artifacts in the DTI dataset were corrected using eddy tool in FSL. After skull-stripping using the Brain Extraction Tool (BET), a diffusion tensor model for each subject was fitted to the data by calculating diffusion tensor parameters using the FMRIB Diffusion Toolbox (FDT). The tract-based spatial statistics (TBSS) tool available in FSL [described in detail previously (Smith et al., 2006)] was used separately on each time point for the complete cohort to calculate the skeletonized mean FA images. First, FA images were normalized using the nonlinear registration algorithm in FSL. The individual normalized FA masks for each subject were then projected onto the TBSS skeleton of the PSMD marker tool to derive the individual skeletonized FA masks. These were then used to extract the mean whole-brain skeletonized FA value of each participant (excluding the cerebellum). FA is dimensionless and assumes values between 0 and 1. Higher FA values indicate higher anisotropy reflecting a higher structural integrity of white matter. The workflow described here was wrapped with R 4.1.1 (R Core Team, 2021).

Volumes of white matter hyperintensities were calculated using BIANCA which is implemented in FSL (Griffanti et al., 2016). We used 121 manually delineated FLAIR images and a training sample of 40 subjects for the training of BIANCA to adapt the program to the challenge of low volume extraction (Wulms et al., 2022). The WMH volume was extracted choosing a threshold of 0.8.

### Statistical Analyses

Relative WMH values were calculated by dividing the WMH volume by white matter (WM) volume. Categorical variables used in the regression analyses described below were included in the models as factors and reference categories were revealed in the corresponding tables. As statistical significance level we used a two-tailed alpha of 0.05.

All statistical analyses were performed with R 4.1.0 (R Core Team, 2021) and RStudio Version 1.4.1717 (RStudio Team, 2021).

#### Association of Sex With White Matter Pathology

The logarithm of relative and absolute WMH values approximately followed a normal distribution. We therefore used a generalized linear model with Gamma distribution and log-link function to assess the age-adjusted association of sex with WMH volumes. Ordinary least squares (OLS) regressions were conducted to obtain the age-adjusted association of sex with FA. Age was included linearly in the models.

#### Association of Vascular Risk Factors With Markers of White Matter Pathology

To examine the association of potential risk factors and markers of white matter pathology (absolute WMH, rel. WMH, and FA, respectively), main effects models, including sex, age (linear, in years), education, smoking, hypertension, diabetes, and obesity, were built. The model for absolute WMH was additionally adjusted for intracranial volume (ICV). To identify the moderation effect of sex, interaction models were conducted separately for each risk factor by including an interaction term between sex and each risk factor. These analyses were controlled for all other risk factors listened above. Additionally, sex-stratified regression models were built. Since WMH volumes approximately showed a log-normal distribution, generalized linear models with Gamma distribution and log-link function were used. For FA, an OLS regression analysis was performed.

#### Association of Magnetic Resonance Imaging Markers With Cognitive Functions

To examine the association of markers of white matter pathology with cognitive functions, sex-stratified OLS regression analyses were performed for every cognitive test (*Z*-Score). Analyses were adjusted for age (natural spline with 2 df) and education. Analyses were repeated and adjusted for age (natural spline with 2 df), education, smoking, hypertension, diabetes, obesity, and CES-D score (</≥16).

## Results

**FIGURE 1:**
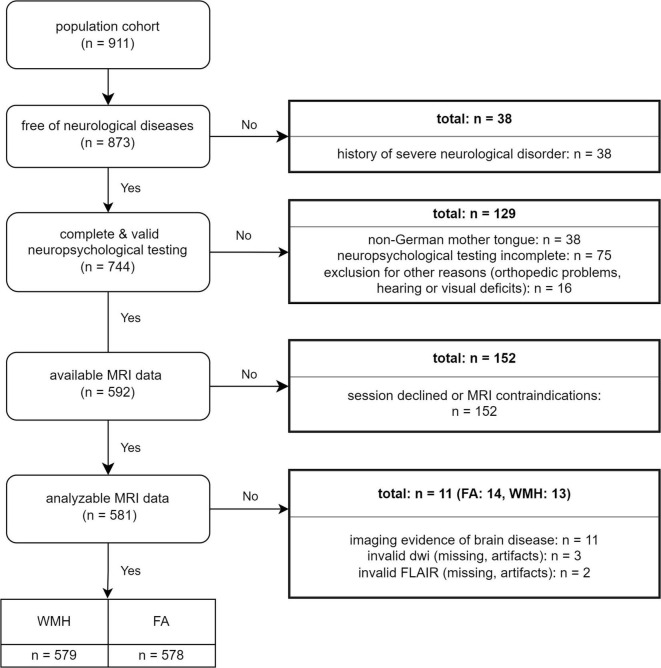
Flow chart.

Table 1 shows the characteristics of our study population. In all, 265 men and 316 women aged 35–66 years were included (Figure 1). From these participants, 92 men (35%) and 71 women (22%) had an untreated and uncontrolled hypertension and 11 (4%) men, and 11 women (3%) had a diagnosed diabetes mellitus. A high CES-D score ≥16 could be observed in 33 men (12%) and 65 women (21%) and obesity in 55 men (21%) and 68 women (22%). Additionally, 53 (20%) men and 63 (20%) women were current smokers.

**TABLE 1:** Characteristics of study participants.

	Total *N* = 581 (100%)	Men *N* = 265 (46%)	Women *N* = 316 (54%)
**Baseline**			
*Age (years)*			
Median (range)	53.1 (35.2–66.2)	52.5 (35.3–66.2)	53.5 (35.2–66.1)
*Hypertension, N* (%)			
Untreated, controlled	282 (49%)	108 (41%)	174 (55%)
Untreated, uncontrolled	163 (28%)	92 (35%)	71 (22%)
Treated, controlled	51 (9%)	22 (8%)	29 (9%)
Treated, uncontrolled	85 (15%)	43 (16%)	42 (13%)
*Diabetes, N* (%)			
No	559 (96%)	254 (96%)	305 (97%)
Yes	22 (4%)	11 (4%)	11 (3%)
*CES-D, N* (%)			
<16	483 (83%)	232 (88%)	251 (79%)
≥16	98 (17%)	33 (12%)	65 (21%)
*Education*, *N* (%)			
Primary or general secondary school	105 (18%)	51 (19%)	54 (17%)
Intermediate secondary school	124 (21%)	35 (13%)	89 (28%)
High school	103 (18%)	46 (17%)	57 (18%)
University graduates	249 (43%)	133 (50%)	116 (37%)
*Smoking status*, *N* (%)			
Never	249 (43%)	112 (42%)	137 (43%)
Former	216 (37%)	100 (38%)	116 (37%)
Current	116 (20%)	53 (20%)	63 (20%)
*Obesity*, *N* (%)			
No, BMI < 30 kg/m^2^	458 (79%)	210 (79%)	248 (78%)
Yes, BMI ≥ 30 kg/m^2^	123 (21%)	55 (21%)	68 (22%)
*Brain volume (ml)*			
Arithm. mean	1455	1550	1375
Range	1000–1906	1281–1906	1000–1758
*Relative brain volume (% of ICV)*			
Arithm. mean	75.80	75.10	76.39
Range	61.63–84.75	61.63–84.38	65.59–84.75
*Abs. WMH (ml)*			
*N*	579	264	315
Geom. mean	0.674	0.682	0.668
Range	0.011–26.406	0.029–22.442	0.011–26.406
*Rel. WMH (% of WM volume)*			
Geom. mean	0.131	0.124	0.138
Range	0.003–4.701	0.006–4.013	0.003–4.701
*FA*			
*N*	578	262	316
Arithm. mean	0.359	0.358	0.360
Range	0.275–0.417	0.275–0.417	0.292–0.409
*Word list (Z-score)*			
Mean (SD)	−0.22 (0.92)	−0.48 (0.9)	0 (0.87)
*Pegboard (Z-score)*			
Mean (SD)	−0.20 (0.94)	−0.44 (0.97)	0 (0.87)
*CWIT, interference time (Z-score)*			
Mean (SD)	−0.09 (1)	−0.19 (0.99)	0 (1)
*TMT A (Z-score)*			
Mean (SD)	−0.01 (1.02)	−0.02 (1.04)	0 (1)
*TMT B (Z-score)*			
Mean (SD)	−0.04 (1.02)	−0.09 (1.04)	0 (1)
*Word fluency test (Z-score)*			
Mean (SD)	0.01 (1)	0.03 (1)	0 (1)

*ICV, intracranial volume; WM, white matter; WMH, white matter hyperintensities; SD, standard deviation.*

### Sex-Differences in Markers of White Matter Pathology

**FIGURE 2:**
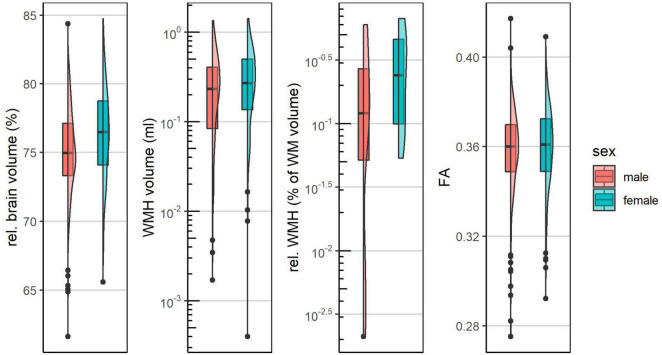
Distributions of MRI markers stratified by sex.

Distributions of MRI markers are shown in Figure 2 and Table 1. The age-adjusted associations of sex with MRI markers are shown in Table 2. Absolute WMH volumes showed a geometric mean of 0.682 ml (range: 0.029–22.442 ml) for men and geometric mean of 0.668 ml (range: 0.011–26.406 ml) for women. The age adjusted association of sex with absolute WMH values was not statistically significant ($\hat{\beta }$ for female sex = 0.025, *p* = 0.824). Relative WMH volumes were lower in men (geometric mean: 0.124% of WM volume) than in women (geometric mean: 0.138% of WM volume), with an age-adjusted association of $\hat{\beta }$ (women) = 0.151 and *p* = 0.171. For FA, a mean value of 0.358 (range: 0.275–0.417) for men and 0.360 (range: 0.292–0.409) for women could be observed. Adjusted for age, female sex was associated with higher FA values ($\hat{\beta }$ = 0.003, *p* = 0.061).

**TABLE 2:** Associations (main effects) of risk factors with MRI markers.

	Rel. WMH^1^ (*N* = 579)	Abs. WMH^1,2^ (*N* = 579)	FA^3^ (*N* = 578)
			
	$\hat{\beta }$ (*p*-value)	$\hat{\beta }$ (*p*-value)	$\hat{\beta }$ (*p*-value)
**Model 1**			
*Constant*	−4.79*** (<2e-16)	−3.051*** (3.19e-15)	0.406*** (<2e-16)
*Sex (Ref: Male)*			
Female	0.151 (0.171)	0.025 (0.824)	0.003 (0.061)
Age (In years)	0.061*** (<2e-16)	0.061*** (<2e-16)	−0.001*** (<2e-16)
**Model 2** ^4^			
*Constant*	−4.189*** (<2e-16)	−4.550*** (8.42e-10)	0.402*** (<2e-16)
*Sex (Ref: Male)*			
Female	0.100 (0.321)	0.228 (0.063)	0.002 (0.154)
Age (In years)	0.050*** (1.53e-13)	0.048*** (3.06e-12)	−0.001*** (8.03e-16)
*Smoking (Ref: Never)*			
Former	−0.004 (0.969)	−0.024 (0.830)	−0.002 (0.224)
Current	0.221 (0.099)	0.213 (0.118)	−0.005* (0.014)
*Diabetes (Ref: No)*			
Yes	0.207 (0.431)	0.237 (0.374)	−0.004 (0.264)
*Hypertension (Ref: Untreated, controlled)*			
Untreated, uncontrolled	−0.127 (0.293)	−0.134 (0.273)	−0.003 (0.057)
Treated, controlled	0.371* (0.049)	0.401* (0.037)	−0.006* (0.030)
Treated, uncontrolled	0.419** (7.71e-3)	0.434** (6.56e-3)	−0.008*** (3.86e-4)
*Obesity (Ref: No)*			
Yes	0.204 (0.105)	0.195 (0.128)	0.0002 (0.900)

*p < 0.05, **p < 0.01, ***p < 0.001.

*^1^Results from generalized linear models with Gamma distribution and log-link function.*

*^2^Additionally adjusted for intracranial volume in Model 2.*

*^3^Results from ordinary least squares (OLS) regression analysis.*

*^4^Additionally adjusted for education.*

### Association of Risk Factors With Markers of White Matter Pathology

The main effects models (Table 2) showed significant positive associations of age with WMH (rel. WMH: $\hat{\beta }$ = 0.050, *p* = 1.53e-13, abs. WMH: $\hat{\beta }$ = 0.048, *p* = 3.06e-12) and a negative association with FA ($\hat{\beta }$ = −0.001 *p* = 8.03e-16). Additionally, hypertension was positively associated with rel. WMH (treated, controlled: $\hat{\beta }$ = 0.371, *p* = 0.049; treated, uncontrolled: $\hat{\beta }$ = 0.419, *p* = 7.71e-3), abs. WMH (treated, controlled: $\hat{\beta }$ = 0.401, *p* = 0.037; treated, uncontrolled: $\hat{\beta }$ = 0.434, *p* = 6.56e-3) and negatively associated with FA (treated, controlled: $\hat{\beta }$ = −0.006, *p* = 0.030; treated, uncontrolled: $\hat{\beta }$ = −0.008, *p* = 3.86e-4). Current smoking was negatively associated with FA ($\hat{\beta }$ = −0.005, *p* = 0.014).

Table 3 shows the moderation effect of sex. For relative WMH the interaction of female sex with former smoking was $\hat{\beta }$ = 0.426 (*p* = 0.039) and with current smoking $\hat{\beta }$ = 0.791 (*p* = 1.76e-3). Similar results could be found for absolute WMH volumes (female sex, former smoking: $\hat{\beta }$ = 0.407, *p* = 0.051; female sex, current smoking: $\hat{\beta }$ = 0.851, *p* = 8.19e-4). The interaction of female sex with a treated and controlled hypertension resulted in $\hat{\beta }$ = 0.634 (*p* = 0.071) for rel. WMH and $\hat{\beta }$ = 0.649 (*p* = 0.069) for abs. WMH.

**TABLE 3:** Moderation effects of sex on the association of potential risk factors with WMH and FA.

	Rel. WMH^1^ (*N* = 579)	Abs. WMH^1,2^ (*N* = 579)	FA^3^ (*N* = 578)
			
	$\hat{\beta }$ (*p*-value)	$\hat{\beta }$ (*p*-value)	$\hat{\beta }$ (*p*-value)
*Sex × smoking*			
Women; former	0.426* (0.039)	0.407 (0.051)	−0.001 (0.729)
Women; current	0.791** (1.76e-3)	0.851*** (8.19e-4)	0.003 (0.367)
*Sex × diabetes*			
Women; yes	0.434 (0.391)	0.392 (0.447)	−0.008 (0.251)
*Sex × hypertension*			
Women; untreated, uncontrolled	0.061 (0.793)	0.052 (0.823)	−0.004 (0.266)
Women; treated, controlled	0.634 (0.071)	0.649 (0.069)	−0.006 (0.225)
Women; treated, uncontrolled	0.438 (0.125)	0.399 (0.169)	−0.006 (0.144)
*Sex × obesity*			
Women; yes	0.106 (0.660)	0.102 (0.676)	−0.004 (0.211)

*p < 0.05, **p < 0.01, ***p < 0.001.

*A separate model was built for each interaction. All results were adjusted for the main effects age, sex, smoking, diabetes, hypertension, obesity, and education.*

*^1^Results from generalized linear models with Gamma distribution and log-link function.*

*^2^Additionally adjusted for intracranial volume.*

*^3^Results from ordinary least squares (OLS) regression analysis.*

Table 4 shows the association of cardiovascular risk factors with MRI markers stratified by sex (abs. WMH, rel. WMH, and FA, respectively). Higher age was associated with higher WMH volumes and lower FA for both, men and women. Current smoking was associated with higher WMH volumes in women (rel. WMH: $\hat{\beta }$ = 0.513, *p* = 0.004, abs. WMH: $\hat{\beta }$ = 0.530, *p* = 0.003). Additionally, hypertension was associated with higher relative WMH volumes in women (treated and controlled: $\hat{\beta }$ = 0.538, *p* = 0.024, treated and uncontrolled: $\hat{\beta }$ = 0.576, *p* = 0.007), higher absolute WMH volumes in women (treated and controlled: $\hat{\beta }$ = 0.568, *p* = 0.019, treated and uncontrolled: $\hat{\beta }$ = 0.580, *p* = 0.007) and lower FA in women (untreated and uncontrolled: $\hat{\beta }$ = −0.005, *p* = 0.035, treated and controlled: $\hat{\beta }$ = −0.009, *p* = 0.015, treated and uncontrolled: $\hat{\beta }$ = −0.011, *p* = 4.03e-4).

**TABLE 4:** Associations of risk factors with MRI markers. Results from sex-stratified analysis.

	Rel. WMH^1^		Abs. WMH^1,2^		FA^3^	
	Men (*N* = 264)	Women (*N* = 315)	Men (*N* = 264)	Women (*N* = 315)	Men (*N* = 262)	Women (*N* = 316)
						
	$\hat{\beta }$ (*p*-value)	$\hat{\beta }$ (*p*-value)	$\hat{\beta }$ (*p*-value)	$\hat{\beta }$ (*p*-value)	$\hat{\beta }$ (*p*-value)	$\hat{\beta }$ (*p*-value)
*Constant*	−3.658*** (1.77e-12)	−4.622*** (<2e-16)	−4.335*** (8.0e-06)	−4.730*** (3.15e-07)	0.400*** (<2e-16)	0.405*** (<2e-16)
*Age (in years)*	0.050*** (4.90e-08)	0.053*** (4.71e-09)	0.048*** (1.2e-07)	0.051*** (2.52e-08)	−0.001*** (7.7e-09)	−0.001*** (2.4e-08)
*Smoking (Ref: Never)*						
Former	−0.216 (0.163)	0.156 (0.266)	−0.227 (0.143)	0.135 (0.343)	−0.002 (0.543)	−0.002 (0.312)
Current	−0.306 (0.104)	0.513** (0.004)	−0.350 (0.062)	0.530** (0.003)	−0.006 (0.058)	−0.003 (0.211)
*Diabetes (Ref: No)*						
Yes	−0.041 (0.909)	0.273 (0.437)	−0.004 (0.992)	0.286 (0.420)	−0.001 (0.904)	−0.007 (0.147)
*Hypertension (Ref: untreated, controlled)*						
Untreated, uncontrolled	−0.146 (0.359)	−0.048 (0.770)	−0.150 (0.344)	−0.054 (0.744)	−0.001 (0.583)	−0.005* (0.035)
Treated, controlled	0.145 (0.591)	0.538* (0.024)	0.179 (0.501)	0.568* (0.019)	−0.003 (0.463)	−0.009* (0.015)
Treated, uncontrolled	0.162 (0.445)	0.576** (0.007)	0.191 (0.363)	0.580** (0.007)	−0.004 (0.206)	−0.011*** (4.03e-4)
*Obesity (Ref: No)*						
Yes	0.151 (0.394)	0.182 (0.258)	0.143 (0.416)	0.173 (0.289)	0.002 (0.516)	−0.001 (0.766)

*Results from sex-stratified analysis.*

*p < 0.05, **p < 0.01, ***p < 0.001.

*^1^Results from generalized linear models with Gamma distribution and log-link function, additionally adjusted for education.*

*^2^Additionally adjusted for intracranial volume.*

*^3^Results from ordinary least squares (OLS) regression analysis, additionally adjusted for education.*

### Association of Magnetic Resonance Imaging Markers With Cognitive Functions

The associations of markers of white matter pathology with cognitive test results are shown in Table 5. Adjusted for age and education, no significant association of any of the cognitive test results (Z-scores) were found with WMH, but higher FA values were positively associated with the Pegboard Test in men ($\hat{\beta }$ = 10.661, *p* = 5.08e-4) and women ($\hat{\beta }$ = 7.064, *p* = 0.012) and with the TMT A and B in men (TMT A: $\hat{\beta }$ = 12.068, *p* = 4.92e-4, TMT B: $\hat{\beta }$ = 7.890, *p* = 0.018). These results remain significant when adjusting for further potential risk factors, with exception of the TMT B in men (*p* = 0.053 after multiple adjustments).

**TABLE 5:** Association of WMH and FA, respectively, with cognitive test results (*Z*-scores).

	Word list	Pegboard	CWIT, interference time	TMT A	TMT B	Word fluency
						
	$\hat{\beta }$ (*p*-value)	$\hat{\beta }$ (*p*-value)	$\hat{\beta }$ (*p*-value)	$\hat{\beta }$ (*p*-value)	$\hat{\beta }$ (*p*-value)	$\hat{\beta }$ (*p*-value)
**Rel. WMH in % (adjusted for age and education)**						
Men (*N* = 264)	−0.140 (0.305)	−0.001 (0.995)	0.051 (0.740)	0.025 (0.878)	−0.038 (0.806)	−0.071 (0.670)
Women (*N* = 315)	0.093 (0.321)	0.098 (0.302)	0.030 (0.781)	−0.009 (0.935)	−0.073 (0.505)	0.174 (0.115)
**FA (adjusted for age and education)**						
Men (*N* = 262)	0.684 (0.815)	10.661*** (5.08e-4)	2.569 (0.438)	12.068*** (4.92e-4)	7.890** (0.018)	0.328 (0.928)
Women (*N* = 316)	2.456 (0.366)	7.064** (0.012)	5.085 (0.107)	1.224 (0.711)	4.924 (0.122)	0.937 (0.771)
**FA (adjusted for age, education, smoking, diabetes, hypertension, CES-D, obesity)**						
Men (*N* = 262)	0.360 (0.905)	9.926** (0.0013)	3.440 (0.310)	11.493** (0.0012)	6.527 (0.053)	0.052 (0.989)
Women (*N* = 316)	1.458 (0.603)	6.104* (0.035)	4.311 (0.182)	0.145 (0.966)	3.099 (0.344)	0.670 (0.841)

*Results were calculated via linear regression models.*

*p < 0.05, **p < 0.01, ***p < 0.001.

## Discussion

The present study reveals interesting sex differences in the association of cardiovascular risk factors and white matter pathology: Women show a lower prevalence of cardiovascular risk factors but a slightly higher burden of white matter damage than men. The impact of hypertension and smoking on white matter was also stronger in women. This leads to the assumption of a higher susceptibility for microvascular damage in women. Regarding potential consequences of white matter damage, we observed no association of WMH with any of the cognitive tests. There was, however, an association of FA with motor functions in both sexes and with executive functions in men only, indicating a higher cognitive reserve in women.

### Sex-Differences in White Matter Pathology

Relative WMH volumes were lower in men (geometric mean: 0.124% of WM volume) than in women (geometric mean: 0.138% of WM volume). Though just not statistically significant on the individual level we observed a pattern of more severe white matter damage in women compared to men comprising slightly higher WMH volumes and lower FA values. These findings are largely in line with previous studies in older cohorts reporting a higher WMH-load in women (Leeuw et al., 2002; van den Heuvel et al., 2004; Wen and Sachdev, 2004; Sachdev et al., 2009; Fatemi et al., 2018; *Alqarni et al., 2021*).

### Sex-Differences in the Association of Risk Factors With White Matter Pathology

Interestingly, we found no significant main effect of sex on WMH or FA, respectively. Significant main effects emerged for age on all white matter lesion phenotypes, current smoking on FA, and treated but uncontrolled hypertension as well as treated and controlled hypertension for WMH and FA. The interaction as well as the stratified analyses showed that current smoking was associated with higher WMH volumes only in women, whereas there was a non-significant trend toward an opposite effect for men. We also observed a more negative impact of hypertension on MRI markers in women. These findings are in line with current evidence that shows hypertension and smoking to be predictors of white matter damage (Dickie et al., 2016). However, the few studies examining sex-difference in the associations of cardiovascular risk factors with white matter pathology show conflicting results regarding sex-preferences as well as potential risk factors. Hypertension has mainly been shown to have a greater impact on WMH in men (Sachdev et al., 2009; Assareh et al., 2014; Filomena et al., 2015; Alqarni et al., 2021). Smoking has been associated with higher WMH in women (Sachdev et al., 2009). Other studies did not find any association with hypertension or smoking in neither men nor women, but, for example sex-specific effects of body mass index on deep white matter lesions (Alqarni et al., 2021). Reasons for these inconsistencies might be differing age-ranges, small sample sizes, varying definitions of risk factors and white matter damage, as well as high a co-linearity of risk factors.

From our results, we conclude, that hypertension and smoking seem to be main risk factors of early brain aging particularly in women. Looking at the effect sizes, treated and/or uncontrolled hypertension as well as current smoking in women have a comparable impact on WMH and FA as up to 10 years of age. This high susceptibility for microvascular brain damage in midlife women may be associated with perimenopausal hormonal changes (Leeuw et al., 2001). Reductions in estrogen levels, which play an important role in brain maintenance via promoting cerebral blood flow, protecting against oxidative stress, and stimulating synaptogenesis, may render the brain especially vulnerable to ischemic damage (Leeuw et al., 2001; Toffoletto et al., 2014; Zárate et al., 2017; Russell et al., 2019). In line with these pathophysiological hypotheses, it is well established that female-specific risk factors such as menopause contribute to cognitive impairment and dementia in women (Gannon et al., 2019). Interactions of varying levels of sex hormones with vascular risk factors in brain aging, however, have not been studied in population cohorts so far.

### Sex-Differences in the Association of White Matter Damage With Cognitive Functions

We found no significant association of any of the cognitive test results with WMH. Higher FA values, however, were associated with better motor functions (Pegboard Test) in both men and women as well as with better working memory, and psychomotor speed, respectively (TMT A and B) in men only.

Some of the above discussed studies, that showed associations of arterial hypertension with white matter damage, also reported declines in executive functions associated with the extent of white matter pathology (Sachdev et al., 2009; Debette et al., 2011; Veldsman et al., 2020). However, only one of these studies evaluated sex-differences in this association. In line with our findings, Sachdev et al. found WMH to be associated with reduced processing speed in men only (Sachdev et al., 2009). One potential explanation why men are cognitively more affected by the same extent of white matter damage, might be sex-differences in cognitive reserve or brain reserve, respectively (O’Dwyer et al., 2012; Stern et al., 2020; Subramaniapillai et al., 2021). Especially in premenopausal women, protective effects of estrogen are supposed to play a role in the maintenance of prefrontal cortex function and consecutively in the preservation of executive functions (Keenan et al., 2001; Shanmugan and Epperson, 2014).

The association of Pegboard Test with white matter damage in both sexes is an interesting finding and supports a recent report of fine motor skills to show a strong association with age-related decline and markers of brain aging (Yao et al., 2021). Further studies are warranted to evaluate whether fine motor skills or motor speed can serve as a particularly sensitive test of cognitive and brain aging.

### Strengths and Limitations

Our study is looking at sex-differences in white matter damage from various angles. Based on the hypothesis that white matter pathology does not simply act as a mediator in the association between vascular risk factors and cognitive decline, we differentiated between sex-differences in the impact of risk factors on white matter pathology on the one side and the impact of white matter pathology on cognition on the other side. Also of note is the age spectrum of 35–65 years, as most cohort studies on aging are settled in later life. It must be noted that we did not adjust for multiple testing. Our results are of explorative nature and thus need to be validated in an independent sample before drawing any conclusions (Bender and Lange, 2001). We might also have missed some effects due to lack of power. For example, the sex-differences in the prevalence of white matter pathology were not significant on their own, but the pattern clearly showed more pathology in women compared to men.

### Summary

The present study extends available evidence on sex-differences in the causes, patterns, and consequences of white matter damage. While we found men to have a slightly worse cardiovascular risk profile, women had a larger volume of WMH. Women’s white matter also showed a higher susceptibility to some risk factors, particularly smoking and arterial hypertension. Nevertheless, associations between microstructural white matter integrity and psychomotor speed were only observed in men, suggesting a higher cognitive reserve in women. Future studies with follow-up, multimodal MRI as well as genetic and hormonal data are warranted to evaluate vulnerable age periods and pathophysiological mechanisms that underlie the observed sex-differences and that can support the proposed mechanisms of brain as well as cognitive reserve. Based on these investigations, studies are needed, that evaluate whether a sex-specific modification of the observed risk factors can slow down white matter damage and cognitive aging.

## Data Availability Statement

The original contributions presented in the study are included in the article, further inquiries can be directed to the corresponding author.

## Ethics Statement

The studies involving human participants were reviewed and approved by the Ethics Committee of the University of Münster and Westphalian Chamber of Physicians in Münster, North Rhine-Westphalia, Germany. The participants provided their written informed consent to participate in this study.

## Author Contributions

NB, NW, and HM drafted the manuscript. NB conducted and programmed all statistical analysis. NW applied the MRI data workflow. MD-N applied the MRI data workflow and revised the manuscript for intellectual content. HM came up with the research idea and supervised all analyses. KB (principal investigator of the BiDirect Study) helped substantially in writing and editing of the manuscript. All authors contributed to the article and approved the submitted version.

## Conflict of Interest

The authors declare that the research was conducted in the absence of any commercial or financial relationships that could be construed as a potential conflict of interest.

## Publisher’s Note

All claims expressed in this article are solely those of the authors and do not necessarily represent those of their affiliated organizations, or those of the publisher, the editors and the reviewers. Any product that may be evaluated in this article, or claim that may be made by its manufacturer, is not guaranteed or endorsed by the publisher.
